# Molecular Epidemiology of Human Rhinovirus Infections in Kilifi, Coastal Kenya

**DOI:** 10.1002/jmv.23251

**Published:** 2012-03-16

**Authors:** Clayton O Onyango, Stephen R. Welch, Patrick K Munywoki, Charles N Agoti, Ann Bett, Mwanajuma Ngama, Richard Myers, Patricia A Cane, D.J Nokes

**Affiliations:** 1 KEMRI-Wellcome Trust Research ProgrammeKilifi, Kenya; 2 Health Protection AgencyLondon, United Kingdom; 3 School of Life Sciences, University of WarwickCoventry, United Kingdom

**Keywords:** acute respiratory illness, rhinovirus, RT-PCR, VP4/VP2 protein, genetic sequencing

## Abstract

This study reports pediatric surveillance over 3 years for human rhinovirus (HRV) at the District Hospital of Kilifi, coastal Kenya. Nasopharyngeal samples were collected from children presenting at outpatient clinic with no signs of acute respiratory infection, or with signs of upper respiratory tract infection, and from children admitted to the hospital with lower respiratory tract infection. Samples were screened by real-time reverse transcriptase polymerase chain reaction (real-time RT-PCR) and classified further to species by nucleotide sequencing of the VP4/VP2 junction. Of 441 HRV positives by real-time RT-PCR, 332 were classified to species, with 47% (155) being HRV-A, 5% (18) HRV-B, and 48% (159) HRV-C. There was no clear seasonal pattern of occurrence for any species. The species were present in similar proportions in the inpatient and outpatient sample sets, and no significant association between species distribution and the severity of lower respiratory tract infection in the inpatients could be determined. HRV sequence analysis revealed multiple but separate clusters in circulation particularly for HRV-A and HRV-C. Most HRV-C clusters were distinct from reference sequences downloaded from GenBank. In contrast, most HRV-A and HRV-B sequences clustered with either known serotypes or strains from elsewhere within Africa and other regions of the world. This first molecular epidemiological study of HRV in the region defines species distribution in accord with reports from elsewhere in the world, shows considerable strain diversity and does not identify an association between any species and disease severity.

## INTRODUCTION

Human rhinoviruses (HRVs), members of the *Picornaviridae* family [Whitton et al., 2005], are recognized as the most frequent viral agents in humans presenting with symptoms of the common cold [Denny, 1995]. The profile of HRVs has been raised as a result of widening use of sensitive molecular methods of detection compared with in vitro cultivation [Kammerer et al., 1994; Arruda et al., 1997; Pitkaranta et al., 1997; Andeweg et al., 1999; Vesa et al., 2001; Renwick et al., 2007], with an increase in the observations of association with lower respiratory tract infection [Lamson et al., 2006; Lau et al., 2007, 2009; Khetsuriani et al., 2008; McErlean et al., 2008; Linsuwanon et al., 2009; Piralla et al., 2009]. A number of other studies have suggested that respiratory illness, presenting with wheezing, rales and respiratory distress may be associated with HRVs [Camara et al., 2004; Cheuk et al., 2007; Singh et al., 2007; Miller et al., 2007, 2009].

Sensitive RT-PCR and sequencing has been used to type rhinoviruses as HRV-A, HRV-B, and HRV-C, with HRV-C being discovered as recently as 2006 [Lau et al., 2007; McErlean et al., 2007]. These PCR methods have also enabled studies on HRV disease burden in hospitalized infants and children under 5 years old [Midulla et al., 2010; Miller et al., 2009, 2007], including sub-Saharan Africa [Niang et al., 2010; O,Callaghan-Gordo et al., 2011; Venter et al., 2011]. Molecular studies suggest almost equal prevalence of HRV-A and HRV-C with similar global distribution patterns [reviewed in Simmonds et al., 2010]. Although most of the epidemiological studies on HRV-C have focused on hospitalized or asthmatic children, the clinical outcomes of HRV-C infection, which was initially controversial, shows association with severe disease including asthma and acute wheezing [McErlean et al., 2007; Renwick et al., 2007; Miller et al., 2009; Peltola et al., 2009; Piralla et al., 2009; Smuts et al., 2011].

HRVs are non-enveloped viruses with an icosahedral capsid enclosing a single-stranded, positive sense RNA genome that is translated in the cytoplasm [Rueckert, 1996]. The viral polyprotein is divided into P1, P2, and P3 regions, with the P1 region encoding the capsid proteins VP4, VP2, VP3, and VP1, while the P2 and P3 regions encode non-structural proteins 2APro, 2B, 2C, 3A, 3B (VPg), 2CPro and 3DPol [reviewed by Kirchberger et al., 2007]. Sequencing of the VP1 led to the classification of HRVs into three species (HRV-A-C) [Ledford et al., 2004] and recently, use of the VP4/VP2 junction, which is less variable and more easy to amplify than VP1, has replicated the earlier VP1 genetic classification [Wisdom et al., 2009; Piralla et al., 2011].

There is little information on the molecular epidemiology of HRVs in sub-Saharan Africa [Peltola and Ruuskanen, 2008]. A recent study associated clinical disease with HRV infection in 58.2% of hospitalized children, most of whom were under 2 years of age (72%), with species distribution of 37% HRV-A, 11% HRV-B, and 52% HRV-C [Smuts et al., 2011] in South Africa. The lack of adequate information on the molecular epidemiology of HRV in tropical sub-Saharan Africa may be attributable to the presumed mild nature of the illness, scarcity of molecular laboratories, unreliably in vitro culture methods [Jartti et al., 2004; Kaiser et al., 2006], and the impracticality of sero-diagnosis due to the large numbers (>100) of identified serotypes [Gwaltney et al., 1966; Johnston et al., 1993].

In this study, the VP4/VP2 junction of HRV samples which were collected from children attending Kilifi District Hospital, coastal Kenya between the years 2007 and 2009 was sequenced. These samples were taken from children presenting with a range of respiratory illness severity, from very severe lower respiratory tract infection to those with and without upper respiratory tract illness. The information on HRV species distribution in rural coastal Kenya, the seasonal prevalence of such species as well as the sequence database of African HRV will increase the global knowledge base of these viruses.

## MATERIALS AND METHODS

### Patients and Samples

The study was undertaken with samples from children presenting to the Kilifi District Hospital, which is located on the coast of Kenya approximately 60 km from Mombasa city. The District comprises a largely rural population of subsistence farmers and experiences an equatorial climate with rains predominantly falling in the months of April to July and November to December. Further details of the study area and respiratory virus disease surveillance through Kilifi District Hospital can be found in previous reports [Nokes et al., 2009; Berkley et al., 2010]. Nasopharyngeal wash or aspirate residues collected from 1912 children aged 1 day—12 years between January 2007 and December 2009, and stored in viral transport medium at -80°C, were tested for the presence of HRV. The children were outpatients (254) presenting either for routine immunization and categorized as without respiratory infection or with signs and symptoms of upper respiratory tract infection [Berkley et al., 2010], or inpatients (1,759) with signs and symptoms consistent with lower respiratory tract infection and categorized as either mild, severe or very severe following WHO syndromic criteria [Nokes et al., 2008]. Written informed consent was obtained from the parent/guardian. This study was approved by the Kenya National Ethical Review Committee and the University of Warwick Biomedical Research Ethics Sub-Committee.

### RNA Extraction and PCR

RNA was extracted from HRV positive samples using the Qiagen Viral RNA miniprep kit (Qiagen, West Sussex, UK) following the manufacturer,s instructions. Diagnostic PCR to confirm the presence of HRV genetic material was performed using a real-time RT-PCR with primers and probe targeting the 5, un-translated region [Hammitt et al., 2011]. For VP4/VP2 amplification, one tube RT-PCR was performed using Qiagen OneStep RT-PCR kit. Primers amplifying VP4/VP2 [Wisdom et al., 2009] were used at a concentration of 1 µM each per reaction in a total volume of 25 µl. PCR products were purified using Qiagen PCR purification Kit and sequenced with both the outer and inner primers in a BigDye chain terminator reaction and analyzed in a ABI 3130xl instrument.

### Sequence Analysis and Phylogenetics

The raw sequences were edited using BioEdit software, translated in Lasergene (DNAStar) and phylogenetic analysis performed with MEGA 5.01 software [Tamura et al., 2011]. The evolutionary history was inferred using Neighbor-Joining method [Saitou and Nei, 1987] using 1,000 bootstrap replicates [Felsenstein, 1985]. Evolutionary distances were computed using the Kimura 2-parameter [Kimura, 1980]. HRV typing of the samples was determined by comparison with reference sequences from GenBank encoding VP4/VP2 protein. Intra-species patristic distances (p-distances) were tested by Neighbor-Joining method using 2,000 bootstrap replicates in MEGA 5.01 software, together with sequences downloaded from http://www.picornaviridae.com/news.htm (Tables SI and SII). Strains as used in this study refer to those reference sequences identified genetically without supporting serotypic information. Samples were considered to form a monophyletic grouping if they had a bootstrap value ≥ 90%. Statistical analyses were done in Stata version 11.0 (StataCorp LP, College Station, TX, www.stata.com) and graphs drawn in GraphPad Prism version 5.01 for Windows (GraphPad Software, San Diego, CA, www.graphpad.com”). Fisher,s exact test was used to test for independence of two categorical variables, while Spearmans correlation was used to test the associations between HRV and climatic conditions.

## RESULTS

Diagnostic real-time PCR identified 23% (441/1,912) of samples as HRV positive. Of these 86% (380/441) were from inpatient children with lower respiratory tract infection, while 14% (61/441) were from outpatient children. The median age (IQR) of non-acute respiratory infection, upper respiratory tract infection, mild lower respiratory tract infection, severe lower respiratory tract infection, and very severe lower respiratory tract infection children was 3.4 months (m) (3.2–11.8 m), 10.4 m (2.0–16.6 m), 18.8 m (9.5–26.9 m), 9.1 m (3.2–19.0 m), and 7.1 m (1.7–23.3 m) respectively. In general, the prevalence of HRV in inpatients was 22% (380/1,759) and 24% (61/254) in outpatients.

### Distribution of HRV Species

The VP4/VP2 region of the genome was amplified from the 441 HRV positive samples with 75% (332/441) success. While 25% (109) samples in total were un-classified to species, the proportions differed significantly between outpatients and inpatients (44% (27/61) vs. 22% (82/380)), as shown in Table I (*P* < 0.001). In the un-classified category, 90% (98/109) failed to amplify and 10% (11) had poor sequence readout. Of the 98 samples that failed to amplify, 65% (69) had late cycle threshold (Ct) values (above 30 cycles), within the real-time diagnostic assay. The distribution of HRV in 298 classified inpatient samples was 47% (141) HRV-A, 4.4% (13) HRV-B, and 48% (144) HRV-C; this distribution did not differ significantly by lower respiratory tract infection severity (*P* = 0.175). In the outpatients, of 34 classified viruses, the proportion of HRV-A was 41%, HRV-B 15%, HRV-C 44% and the distribution did not differ significantly between children with and without upper respiratory tract infection (*P* = 0.221).

**Table 1:** HRV Species Distribution and Proportion Un-Classified From Pediatric (Under 12 Years of Age) Cases Identified at Kilifi District Hospital Outpatient Clinic and Inpatient Wards 2007–2009

Category	HRV-A	HRV-B	HRV-C	Un-typed	Total
Non-ARI	8 (34.78)	1 (4.35)	4 (17.39)	10 (43.48)	23
URTI	6 (15.79)	4 (10.53)	11 (28.95)	17 (44.74)	38
Mild	18 (43.9)	2 (4.88)	15 (36.59)	6 (14.63)	41
Severe	96 (34.91)	7 (2.55)	111 (40.36)	61 (22.18)	275
V. Severe	27 (42.19)	4 (6.25)	18 (28.12)	15 (23.44)	64
Total	155 (35.15)	18 (4.08)	159 (36.05)	109 (24.72)	441

URTI, upper respiratory tract infection; ARI, acute respiratory illness; un-typed, unclassified.

### Seasonal Distribution of the HRV Species

**Fig 1:**
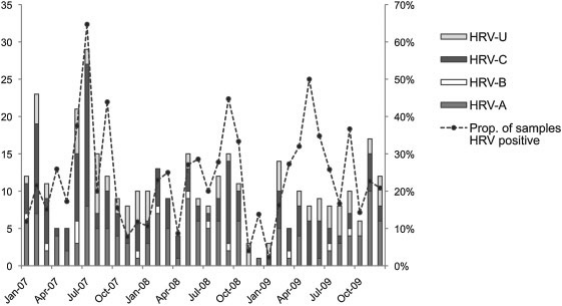
The monthly distribution of HRV species over a 3-year period diagnosed in children admitted with lower respiratory tract infection to Kilifi District Hospital, 2007–2009. Also included on the right *y*-axis are the proportions of the samples from inpatients with lower respiratory tract infection that were HRV positive.

Cases of HRV were identified in Kilifi District Hospital throughout the year over the 3-year study period with higher proportions being seen in the months February 2007, June–July in 2007, August–September 2008, February 2009, April–June 2009, and November 2009, while lower proportions were observed between October–November 2007 and November–December 2008 (Fig. 1).

**Fig 2:**
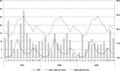
Average daily rainfall (mm, primary *y*-axis) and relative humidity (%, secondary *y*-axis), by month over the period 2007–2009, for Kilifi (source Kilifi Institute of Agriculture) and corresponding monthly HRV cases admitted to Kilifi District Hospital (primary *y*-axis). Note that monthly weather data represent averages for the 3 years.

The distribution of the HRV species and the proportion of samples positive, in the inpatients, over the 3-year period is shown in Figure 1. HRV-A and C co-circulated in most months of surveillance, and while either was rarely absent (e.g., in the months of November 2008–January 2009), the numbers fluctuated considerably on an irregular basis. In contrast, HRV-B occurred only rarely and sporadically throughout the 3 years. Peak admissions of HRV-A and HRV-C cases did not obviously segregate or alternate and were not regularly seasonal. Numbers of HRV-C cases were higher in July 2007, and again in September 2008; while HRV-A cases were higher in July 2007, February and June 2008. There was no clear association between the monthly number of HRV cases and mean relative humidity (r^2^ = 0.1507, *P* = 0.380) or daily mean rainfall (r^2^ = -0.1399, *P* = 0.416) over the period of this study (Fig. 2).

### Relatedness of Kilifi Sequences to Other HRV Sequences

**Fig 3:**
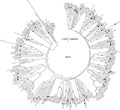
A phylogenetic analysis of HRV-A sequences from Kilifi and reference sequences from Genbank. Sequences with closed circles denote known serotypes, while those with open circles denote previously described strains. Bootstrap values are (2,000 replicates) shown on the branches, with values <90% omitted from the tree. The tree was drawn to scale, with branch lengths in the same units (p-distance) used to infer the phylogenetic tree. Kilifi sequences are designated K-number (KI for inpatient sequences and KO for outpatient sequences).

**Fig 4:**
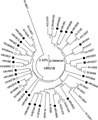
A phylogenetic analysis of HRV-B sequences from Kilifi alongside reference serotypes and strains from Genbank. Sequences with closed circles denote known serotypes, while those with open circles denote previously described strains. The percentage of replicate trees in which the associated taxa clustered together in the bootstrap test (2,000 replicates) is shown next to the branches. Bootstrap values <90% have been omitted from the tree. The tree is drawn to scale, with branch lengths in the same units (p-distance) used to infer the phylogenetic tree. Kilifi sequences are designated K-number (KI for inpatient sequences and KO for outpatient sequences).

**Fig 5:**
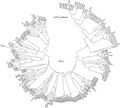
A phylogenetic analysis of HRV-C sequences from Kilifi alongside reference serotypes and strains from Genbank. Sequences with closed circles denote known strains. The percentage of replicate trees in which the associated taxa clustered together in the bootstrap test (2,000 replicates) is shown next to the branches. Bootstrap values <90% have been omitted from the tree. The tree is drawn to scale, with branch lengths in the same units (p-distance) used to infer the phylogenetic tree. Kilifi sequences are designated K-number (KI for inpatient sequences and KO for outpatient sequences).

The Kilifi sequences for each of the species formed numerous unique clusters with mean nucleotide identity of 76.4% (range 52.2%–97.6%) for HRV-A, 74.2% (range 44.5–99.7%) for HRV-B, and 68% (range 51.0–98.9%) for HRV-C, suggesting considerable diversity in strains/serotypes present in the population. In the cases of HRV-A and HRV-B most sequences clustered with known serotypes or strains from around the world (Figs. 3 and 4). In contrast, many of the sequences of HRV-C identified in circulation in Kilifi (Fig. 5) did not cluster with any published strains that also are based on sequencing of the VP4/VP2 junction. The Kilifi sequences (most easily seen for HRV-A and HRV-B) showed reasonable genetic deviation from nearest known strains/serotypes, generally with mean nucleotide differences of 25% (range 15.1–48.0%) for all the three HRV species.

One sequence (KI-5607), that seemed an offshoot in HRV-B (Fig. 4) was found to be human enterovirus 68 when compared with other sequences from the GenBank. The KI-5607 had 95% nucleotide identity to strain JPOC10-396 isolated in Osaka Japan with GenBank accession number AB601884.

## DISCUSSION

The distribution of HRV species in sub-Saharan Africa is not well described although there are some reports on the overall prevalence of HRV. Recently, epidemiological studies in sub-Saharan Africa showed the prevalence of HRV in children <5 years presenting to a primary care center with acute respiratory infection as 17% in rural Senegal [Niang et al., 2010], 41% in rural Mozambique [O,Callaghan-Gordo et al., 2011], and 33% in Pretoria South Africa [Venter et al., 2011]. The equivalent prevalence in inpatients from the present study was 22%. The current study reports the species typing of 441 Kilifi HRV positive samples by the genetic analysis of the VP4/VP2 junction. Failure to type 25% of HRV positive samples was largely attributed to PCR failure (65%) due to low viral load (inferred from Ct value) than the severity category. However, it cannot be excluded that certain genetic variants were missed in the typing assay due to their variability in the primer-annealing sites, leading to PCR failure.

All the three species of HRV were found to be circulating in children under 12 years of age in Kilifi, with occurrence of 47% HRV-A, 5% HRV-B, and 48% HRV-C. Such patterns of species distribution have been reported elsewhere in the USA [Miller et al., 2011] and Cape Town, South Africa [Smuts et al., 2011].

The current study also identified a human enterovirus 68, which was diagnosed initially as HRV by real-time PCR assay. This discrepancy in diagnosis was resolved after BLAST search correctly identified the virus. This was also supported by its unique topology on the HRV-B phylogenetic tree (Fig. 4). The identification of non-HRV is not unique as the PCR primers used the Kilifi HRV diagnostic platform are capable of amplifying other non-HRV human enteroviruses [Wisdom et al., 2009].

There was neither a clear seasonal circulation pattern nor an identifiable influence of weather (rainfall and humidity) on HRV occurrence in Kilifi. This is in contrast to a study from Germany, which showed an association between relative humidity and rhinovirus infection in a hospitalized cohort [du Prel et al., 2009].

This study also noted lack of an association between inpatient severity and any of the HRV species. Other studies elsewhere have shown evidence of the involvement of HRV-C in infants and toddlers with wheeze, and asthma in older children [Lau et al., 2007; Khetsuriani et al., 2008; Miller et al., 2009] and a shorter duration of asthma symptoms and less cough in HRV-A infected children [Arden and Mackay, 2010]. The discrepancy in associating HRV-C with disease severity in the current study may be as a result of either the broad case definition used hence masking the broad definitions of non-acute respiratory infection and lower respiratory tract infection or as a result of the emergence of HRV-C strains with altered disease causing potential.

This study did not attempt to compare inpatient and outpatient cases to identify HRV disease severity by species due to the bias in design. It is therefore important that studies in African settings focus on case–control studies as well as collecting adequate clinical history to help decipher the clinical relevance of HRVs.

There was high diversity of sequences in circulation in all the Kilifi HRV species, although sequences of HRV-A and HRV-B clustered with known serotypes and strains. However there were a large number of clusters for HRV-C that had no closely associated strain from the literature. The formation of few clusters between the known HRV-C and Kilifi sequences compared with HRV-A and HRV-B was mostly like due to the availability of few HRV-C VP4/VP2 sequences in the GenBank.
